# Cultural regulation of emotion: individual, relational, and structural sources

**DOI:** 10.3389/fpsyg.2013.00055

**Published:** 2013-02-12

**Authors:** Jozefien De Leersnyder, Michael Boiger, Batja Mesquita

**Affiliations:** Center for Social and Cultural Psychology, Faculty of Psychology and Educational Sciences, University of LeuvenLeuven, Belgium

**Keywords:** emotion regulation, culture, relationships, appraisal, situation selection, structural affordances, cultural differences

## Abstract

The most prevalent and intense emotional experiences differ across cultures. These differences in emotional experience can be understood as the outcomes of emotion regulation, because emotions that fit the valued relationships within a culture tend to be most common and intense. We review evidence suggesting that emotion regulation underlying cultural differences in emotional experience often takes place at the point of emotion elicitation through the promotion of situations and appraisals that are consistent with culturally valued relationships. These regulatory processes depend on individual tendencies, but are also co-regulated within relationships—close others shape people's environment and help them appraise events in culturally valued ways—and are afforded by structural conditions—people's daily lives “limit” the opportunities for emotion, and afford certain appraisals. The combined evidence suggests that cultural differences in emotion regulation go well beyond the effortful regulation based on display rules.

In her ethnography *Never in Anger,* the anthropologist Jean Briggs describes her time with the Utku Inuit (Briggs, 1970). Adult Utku Inuit rarely express anger: The observation that gave the book its name is not hard to understand when you consider how important it is for a group without technological infrastructure to stick together in a cold and unforgiving climate. The group's closeness and harmony and, therefore, the avoidance of anger, was a central cultural goal for the Utku Inuit. Although Briggs describes a few instances of suppression and displacement of angry behavior—hitting the dogs is one such instance—most of the cultural regulation among the Inuit seemed to be focused on avoiding the occurrence of anger. Anger was rare, because there were few anger antecedents, and because few situations were interpreted as such: Utku Inuit avoided frustrating each other, and in addition, they were very slow to interpret someone else's behavior as frustrating.

The example illustrates the phenomenon of *cultural regulation*, which we understand as the combined cultural processes that result in the alignment of emotions with cultural values, ideals, goals and concerns. Particularly, we will argue (1) that emotional experiences tend to be congruent with culturally central values, ideals, goals, and concerns, (2) that regulation towards culturally congruent emotions often takes place at the point of emotion elicitation, and (3) that regulation happens at the levels of individual tendencies, relational co-regulation and structural affordances. We will support these arguments by discussing cross-cultural evidence.

## Patterns of emotional experiences are congruent with culturally valued relationships

### Emotions, emotion regulation, and social relationships

Emotions are central to social relationships (e.g., Jankowiak and Fischer, 1992; Frijda and Mesquita, 1994; Keltner and Haidt, 1999; Oatley et al., 2006; Mesquita, 2010). By having and expressing an emotion, we take a stance in the social world, express our concerns, and reveal our strategies, goals, and intentions to act (Frijda, 1986, 2007; Solomon, 2004; Griffiths and Scarantino, 2009). For instance, when Mary feels guilty, she holds herself responsible for John's unhappiness, she implies that John's wellbeing is important to her, and she is resolved to make up for what she did wrong. In contrast, when Mary is angry at John, she holds him responsible for something bad, she implies that he violated her individual rights or her personal autonomy, and she is intent on confronting him or taking revenge. Having a particular emotion is thus tantamount to engaging in a relationship in a particular way. When Mary feels guilty, her relationship with John is very different from when she feels angry.

To successfully manage our relationships with others, we need to have and express certain emotions. *Emotion regulation* refers to all the processes that help to attain culturally appropriate (or functional)^1^ emotional experiences; appropriate are those experiences that, within a culture, are more often than not instrumental in the successful navigation of the social world. What these experiences are may differ across cultures; yet, universally, emotion regulation appears to be motivated by a person's need to establish and maintain proper and good relationships (Thompson, 1991; Gross et al., 2006).

### Cultural differences in valued relationships

To have proper and good relationships with others means something different in different cultures (e.g., D'Andrade, 1984; Holland and Quinn, 1987; Bruner, 1990; Markus and Kitayama, 1991; Shweder and Haidt, 2000). Given these differences in the valued relationships, we can assume that the emotions that are “helpful” or functional in coordinating people's relationships may differ accordingly. The relationship ideals between European and East Asian cultural contexts may serve as an example.

In European American contexts, a “good relationship” is one in which each partner remains autonomous and partners mutually strengthen each other's individuality and independence (Triandis, 1995; Kim and Markus, 1999; Rothbaum et al., 2000). Individuality is, among others, strengthened by focusing on the positive characteristics that show each partner's uniqueness and that enable them to be self-reliant; hence there is an emphasis on high self-esteem (Hochschild, 1995; Heine et al., 1999). It is important for this kind of relationship that partners are able to take a stance or assert their desires; (constructive) conflict is not eschewed but rather considered a necessary bump in the road to strong relational ties (Canary et al., 1995). Emotions such as pride and anger appear to be functional in European American relationships since they reflect individual self-worth and personal autonomy; shame and guilt, on the other hand, are less valued since they may threaten a positive self-view.

In contrast, “good relationships” in most East Asian cultural contexts are those in which partners are interdependent and interconnected and adjust to each other's expectations (Lebra, 1992; Heine et al., 1999; Kim and Markus, 1999; Oishi and Diener, 2003). In order to meet these relational expectations, individuals need to be aware of and improve on their shortcomings; hence the focus is on negative information about oneself (Kitayama et al., 1997). In East Asian interdependent cultural contexts, emotions such as shame and guilt appear to be conducive to building strong relationships because they highlight flaws and shortcomings and thus promote alignment with social rules and relational embeddedness. In contrast, anger appears to be highly undesirable in interdependent relationships because it may threaten relational harmony; in that sense, East Asian contexts may be similar to the Inuit context described before.

### Culturally different pattern of emotions

In each culture, the “endpoints” of emotion regulation are dictated by the culturally valued relationship models. In the example of the Inuit described in *Never in Anger,* anger avoidance was implicated by the ideal of social harmony. If we assume that people are reasonably successful regulators of emotion, this should result in cross-culturally different emotional experiences: Anger was rarely expressed (and rarely felt) by the Inuit. The low rate of anger feelings occurred notwithstanding the evidence that the Inuit had the potential for anger: They got angry at their dogs at times, and they also ended up being very angry at the ethnographer herself after she had violated the principles of harmony. This reflects a general pattern in ethnological and cross-cultural research: While there are impressive similarities in the potential for emotions, the actual cultural patterns of emotional experience, and thus the endpoints of emotion regulation, differ cross-culturally in meaningful ways (Mesquita et al., 1997). These differences in the actual cultural patterns can be understood from the cultural relationship ideals (e.g., Kitayama et al., 2006; Mesquita and Leu, 2007).

For instance, Kitayama and colleagues investigated the frequency and intensity of different types of emotions in US and Japanese students, using a retrospective self-report study and a diary study (Kitayama and Markus, 2000; Kitayama et al., 2006). *Socially disengaging* emotions—such as pride, anger, or irritation—were found to be more frequent and intense in European American than in Japanese cultural contexts; this is consistent with the European American emphasis on autonomy and independence. In contrast, *socially engaging* emotions—such as close feelings, shame, guilt, or indebtedness—were found to be more frequent and intense in Japanese than in European American cultural contexts; this is in line with the East Asian emphasis on relatedness and interdependence.

That cultures dictate the endpoints of emotion regulation is also suggested by recent studies on emotional acculturation (De Leersnyder et al., 2011). In these studies, we found that immigrants converge to the endpoints of regulation dictated by their host culture. Korean immigrants to the US, and Turkish immigrants to Belgium shared their host cultures' emotional patterns to the degree they had spent time in the new country; immigrants who reported more daily interactions with members of the host culture—European Americans and native Belgians respectively—reported patterns of emotional experience that were more similar to those reported by members of the host cultures. Immigrants' emotional patterns seem to change due to exposure to new relationship ideals, and to the endpoints of emotion regulation that fit these ideals.

Several studies also suggested that attaining the culturally defined endpoints of emotion regulation is rewarding. In the diary study mentioned above (Kitayama et al., 2006), European American and Japanese students reported more positive adjustment when their emotions were closest to the cultural ideal. Specifically, European Americans experiencing disengaging emotions (pride, anger), and Japanese experiencing engaging emotions (friendly, shame) reported the highest wellbeing. In a related study (De Leersnyder et al., in preparation), Belgian students reported on one of four types of situations, defined by valence (positive, negative) and engagement (engaged, disengaged). The students then rated their emotions at the time of the situation on 30 different items. These ratings resulted, for each individual, in a pattern of emotional intensity. Per situation type, we also calculated the average Belgian pattern of emotions. We found support for the idea that the cultural “endpoints” of emotion regulation are socially rewarding: The correlation between a person's patterns and the cultural average of emotional experience was associated with that person's self-reported well-being, as indicated by fewer depressive symptoms and more satisfaction with their social relationships.

In sum, it may be inferred that the culturally valued relationship models dictate the endpoints of emotion regulation, and that attaining these endpoints is rewarding. In the remainder of this review, we discuss two types of antecedent-focused emotion regulation through which this may be achieved: situation selection and appraisal.

## Antecedent-focused emotion regulation as a source of cultural differences

### Antecedent-focused emotion regulation: situation selection and appraisal

Emotion regulation has always been considered an important source of cross-cultural differences in emotions (e.g., Ekman, 1992). Traditionally, emotion regulation was conceived as a conscious effort to suppress or change emotions due to the salience of cultural display rules. For example, in one experiment, Japanese students as compared to European Americans showed fewer negative emotions in response to a disturbing movie when another person was present, but this was not the case when they watched the movie by themselves. Japanese display rules were thought to underlie this difference in the expression of negative emotions (Ekman and Friesen, 1969). Cross-cultural evidence of a much later date provides general support for the notion that collectivist cultures, such as Japan, have display rules of suppression, at least for certain emotions (Matsumoto et al., 2008). Yet, there is no evidence that cultural differences in suppression of emotional responses in fact underlie the culturally different emotional outcomes; it is equally questionable if suppression is the only or even the strongest force in shaping cultural differences in emotions.

In the current article we review evidence for the idea that emotion regulation often occurs during the process of emotion elicitation. Our review is organized around two constituent processes of emotion regulation, namely situation selection and appraisal, that both affect whether an emotion is elicited, and what the nature of the emotion is. “*Situation selection*” has been described as “approaching or avoiding certain people, places, or objects in order to regulate emotions” (Gross, 1998, p. 283); *appraisal* involves taking an evaluative stance (Solomon, 2004). In this review, we provide evidence that both types of emotion regulation produce cultural differences in emotional experience.

We focus on appraisal generally as a site of emotion regulation, rather than limiting the discussion to re-appraisal specifically, as most of the emotion regulation literature does (e.g., Gross, 1998, 2007). The first reason is that, with few exceptions, appraisal and re-appraisal are hard to distinguish both conceptually and empirically (Campos et al., 2004; Mesquita and Albert, 2007; Mesquita and Frijda, 2011), because these processes often occur automatically (Mauss et al., 2008). The second reason is that culture-level regulation may affect the initial appraisal rather than re-appraisal; culture renders certain appraisals more salient than others, thereby “regulating” the emotions that people are likely to experience in their culture.

### Different sources of emotion regulation

The literature on emotion regulation has primarily focused on *individual*-level emotion regulation (Gross, 1998; Gross et al., 2011)—e.g., Mary is angry at John, but she tries to reinterpret his rude behavior by telling herself that he has been under a lot of pressure, or may just have been oblivious to the consequences of his behavior. (Re-)Appraisal at this level may be subject to cultural influence when culturally prevalent ideals about how to relate to (certain) others affect the ways in which individuals appraise situations. For instance, the cultural ideal that a woman should support her husband may make Mary more likely to attribute John's behavior to external pressures—both in terms of her initial appraisal or her later re-appraisal of John's behavior. This is an example of culturally influenced individual-level regulation.

**Figure 1:**
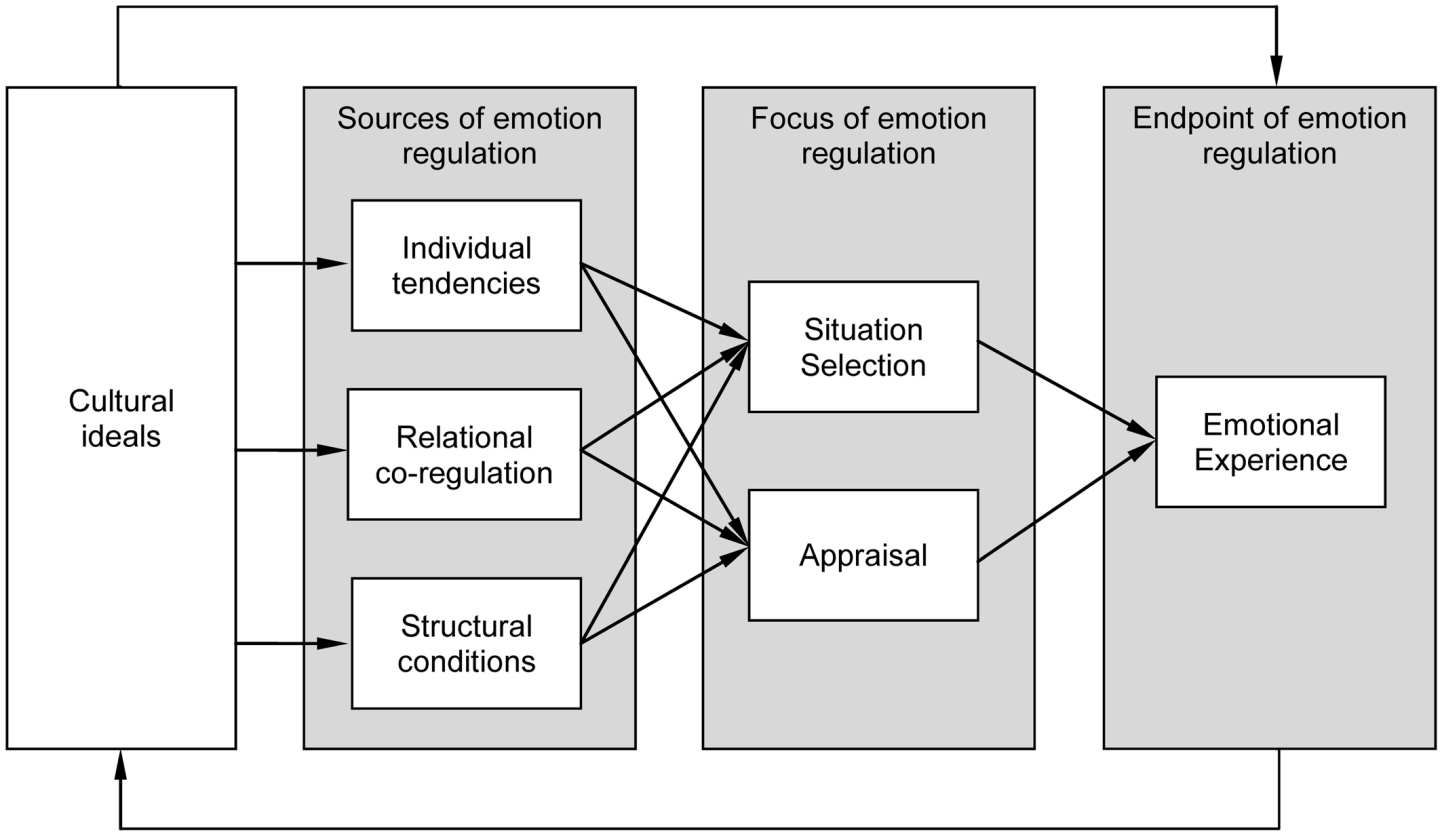
**Three sources of antecedent-focused emotion regulation in cultural context**.

We distinguish two other sources of regulation. First, there is some evidence for *relational* co-regulation by close others, most notably the work on parents' regulation of children's emotions—e.g., a caregiver telling the child that her brother did not break the toy on purpose, and that she should get over her anger (e.g., Eisenberg et al., 1999; Campos et al., 2004; Holodynski and Friedlmeier, 2006). Furthermore, we distinguish a third source of emotion regulation, which is of a *structural* nature: The organization of everyday life affords certain types of emotional situations, and suppresses others. Our review of cultural differences in emotion regulation includes all three sources (individual, relational and structural) for the two types of antecedent-focused emotion regulation (situation selection and appraisal). Figure 1 shows how cultural ideals provide a background against which individual tendencies, relational co-regulation and structural affordances bring about certain emotional experiences through these two types of regulation.

### Situation selection

Mary may avoid seeing John when he is stressed, because she knows his rude behavior would make her angry. This is what has been referred to as situation selection: approaching or avoiding certain people, places, or objects in order to regulate emotions. At a relational level, situation selection may take place when people structure each other's experiences by encouraging one another to avoid or seek out certain situations. For example, in an attempt to avoid anger, the Utku Inuit structured their interactions in ways to avoid confrontations at all costs. The structural organization of everyday life may fulfill a similar role: politeness rules in some societies reduce the likelihood of experiencing anger eliciting encounters (Cohen, 1999). Situation selection may thus take place at the individual, the relational, and the structural level; culture may play a role at all levels.

#### Individual tendencies

People's selection of situations and according emotional experience is, for example, shaped by their motivational focus. A promotion focus leads to happiness in the case of success, and to depression in the case of failure, whereas a prevention focus leads to relief in the case of success, and anxiety in the case of failure (Higgins et al., 1997; Lee et al., 2000). Research on motivational focus suggests that people from cultures that value autonomy and individuality as relationship goals (e.g. US contexts) are more focused on the *accomplishment* of positive outcomes (i.e., promotion focus), whereas people in cultures emphasizing relational harmony and obligation (e.g. East Asian, Russian) are more concerned with *avoiding* negative outcomes (i.e., prevention focus; Lee et al., 2000; Elliott et al., 2001). American respondents thus seek out situations that promise success, whereas East Asians and Russians avoid situations that are likely to lead to failure (for instance, the failure to meet social expectations). A cross-cultural vignette study on success and failure in European American and Chinese cultural contexts confirmed that these cultural differences in individual-level situation selection give rise to differences in emotional experience (Lee et al., 2000). Consistent with their cultural focus on promotion, European Americans reported a higher intensity of happiness/depressed emotions than relief/anxiety. Conversely, consistent with their cultural focus on prevention, the Chinese group reported a higher intensity of relief/anxiety than happiness/depressed emotions. This is some first evidence that the differences in situation selection at the level of approach or avoidance are related to differences in the prevalent types of emotions.

People also tend to seek out situations that elicit culturally “ideal” affect (Tsai, 2007). What is considered ideal affect differs between cultures: European Americans prefer high activation positive emotions (e.g., excitement), because these emotions foreground individual experience and prepare people for asserting themselves and influencing others (Tsai et al., 2007). In contrast, East Asians value low arousal positive states (e.g., calm, relaxed), because these emotional states facilitate attention to the context (Bradley et al., 2001 as cited in Tsai, 2007) and prepare people for adjusting their behavior to others' needs (Tsai et al., 2007). Consistently, survey research has documented cultural differences in the activities that people in the respective cultural contexts seek out: While North Americans seek out active individual activities (e.g., running or rollerblading), up-beat music, and stimulants (e.g., amphetamines, cocaine), East Asians are drawn to passive collective activities (e.g., sightseeing, picnicking), calmer music, and sedatives (e.g., opiates) (Gobster and Delgado, 1992; Tsai, 2007). By selecting certain situations over others, individuals achieve those emotional states that are functional in their respective cultural context.

#### Relational co-regulation

In many ways, situation selection takes place in the context our relationships with others; hence, interactions with others shape our emotional experience (Boiger and Mesquita, 2012). Although people structure each other's emotional lives throughout the lifespan (Mesquita, 2010), this phenomenon is especially apparent in the first years of life. Parents organize their children's lives almost entirely; moreover, parental efforts appear to be in the direction of promoting situations that elicit culturally valued experiences (e.g., Goodnow, 1997; Güngör et al., 2011). By shaping children's environments, parents allow for and highlight certain (emotional) experiences over others; they can thus be said to co-regulate the child's emotional life through situation selection. Across cultures, parents appear to select different situations for their children. In each case, parents' situation selection can be understood as an attempt to align their children's emotional experiences to the culturally desired endpoints of emotion regulation, thus helping their children to successfully navigate their social relationships.

Co-regulation occurs, for instance, when parents highlight or re-activate certain emotional experiences as a learning opportunity for their children. Across different cultures, parents appear to highlight the types of emotional experiences that are central to the culture's relationship ideals (Whiting and Whiting, 1975; Röttger-Rössler et al., in press). For instance, Taiwanese parents believe it is necessary and effective to highlight shame when their pre-school children transgress social rules (Wang, 1992; Fung, 1999; Fung and Chen, 2002 as cited in Fung, 1999). Consistently, Fung (1999) observed that Taiwanese parents of 2.5 year olds engaged their child approximately three times an hour in discussions about shame episodes; the majority of which were still ongoing. Most of these discussions were playful, and served as a tool to teach children right from wrong, rather than to denigrate them. Whenever Taiwanese children were about to transgress a social norm, their parents constructed a discussion in such a way that they would experience shame—an emotional experience that would teach the children how to behave properly. The highlighting of shame is not universal. Observational studies with European American parents in the tough and dangerous lower class neighborhoods of Baltimore provide a sharp contrast to the Taiwanese example (Miller et al., 1996, 1997). The Baltimore parents actively avoided to turn their children's wrongdoings in shameful situations by rarely acknowledging their children's rule violations. In the rare cases when parents did talk about these transgressions, they did not treat them as serious wrongdoings in order to “toughen” their children.

Another way in which parents promote relevant emotional situations is by engaging their children in memory conversations. Parents frequently use these conversations to reminisce about recent emotional events (Ross and Wang, 2010). Reminiscing about these situations may in itself again give rise to culturally valued emotional experiences. In one study, European American and Chinese mothers were asked to discuss recent emotional events with their children (Wang, 2001). European American and Chinese mothers not only differed in the events they chose to discuss, but also in the way they discussed them. While European American mothers focused on personal and non-social events, Chinese mothers discussed events in which other people were involved. The European American mothers engaged in a highly elaborative style, stressing the child's own role in the emotional event. In doing so, they constructed conversational situations in which the child's own emotions and view on the situation were paramount. These situations have likely afforded the experience of disengaging emotions such as pride or anger—at the time of the conversation as well as for future events. In contrast, Chinese mothers were much less likely to elaborate on the child's experience of the event in detail. Instead, they focused on the perspective of others who were involved, as well as on the appropriate social behavior that would have been expected from their children. In doing so, the Chinese mothers conveyed the interdependent nature of emotions, thereby underlining the role of their children's emotions in social interactions and teaching them important lessons about social conventions. Moreover, they encouraged their children to experience emotions that are socially engaging such as respect or shame, both during the conversation as well as for the future.

Relatedly, differences in maternal sensitivity influence how and when mothers intervene in structuring their children's environment, and consequently their emotions. While mothers who display “reactive sensitivity” allow negative situations to happen and restrict their interventions to cases where the child experiences a full-blown emotion, “proactive” mothers monitor their child's surroundings and intervene before a negative emotional situation has had the chance to develop (cf. Trommsdorff and Rothbaum, 2008). The extent to which mothers use proactive versus reactive strategies differs between cultures; for example, German mothers were found to use more reactive strategies than Japanese mothers; the latter focus more on proactive strategies (Trommsdorff and Friedlmeier, 2010). In this study, differences in maternal sensitivity caused the children to have different emotional experiences: The proactive Japanese mothers protected their children from negative experiences by removing or distracting them; in contrast, the reactive German mothers exposed their children to negative experiences. These differences in maternal situation selection are consistent with the culture's view on negative emotions. In a German context, where children need to learn to assert themselves, experiencing and expressing negative emotions may be more acceptable and functional than it is in a Japanese context, where the expression of disengaging negative emotions is seen as a threat to close relationships.

#### Structural conditions

The structure of everyday life can be seen as the selection of situations that habitually happen; this selection renders the experience of certain emotions more or less likely. Everyday life differs across cultures, and prevalent emotional experiences differ accordingly. For example, European American social life is characterized by practices that make individuals feel special and unique; these practices afford happiness and feeling good about one's (independent) self (D'Andrade, 1984; Nisbett, 2003). In comparison, many of the Japanese cultural practices promote shame; this is consistent with the Japanese cultural model that emphasizes self-criticism in order to live up to the expectations of others (Heine et al., 1999). For example, at the end of each day, Japanese school children are encouraged to engage in critical self-reflection (“*hansei*”). This practice highlights shortcomings or weaknesses and encourages improvement (Lewis, 1995), thereby affording emotions such as shame.

These ethnographic observations were confirmed by a cross-cultural study in which European Americans and Japanese were asked to report on their interactions (Kitayama et al., 1997). A different group of European American and Japanese rated these interactions with regard to the self-esteem they would afford. The authors found that the European Americans had reported situations that afforded self-enhancement (in both European Americans and Japanese of the second group), which may have promoted high-activation happiness and pride. On the other hand, the Japanese situations afforded more self-criticism, which may have promoted calmer emotional states, wariness and shame. Everyday Japanese life may thus offer more opportunity to feel ashamed, whereas European American daily life may offer the opportunity to feel pride.

People's emotions appear to hinge indeed on the situations that have been “selected” to occur frequently (Boiger et al., in press, Study 1). In this study, we started from the idea that situations that elicit culturally desirable or condoned emotions should be promoted—and thus occur frequently, while situations that elicit culturally undesirable or condemned emotions should be avoided—and thus occur rarely. In line with the dominant cultural ideals in the US and Japan, we predicted that anger is condoned in the US and condemned in Japan because it highlights personal desires and threatens relational harmony; shame is condemned in the US and condoned in Japan because it highlights personal flaws and emphasizes social conventions. North American and Japanese participants indicated for a number of situations from both cultures how frequently most students they know would encounter these situations and to what extent they would feel the associated emotion (i.e., either anger or shame). In line with our predictions, American students perceived situations as more likely to occur to the extent that they elicited stronger feelings of anger. In contrast, Japanese students, perceived situations as less likely to occur when they were highly angering. The opposite picture emerged for shame: Japanese students rated the situations that elicit stronger feelings of shame to be more likely to occur than American students, who perceived them as rather uncommon. Structural situation selection may account, at least partially, for the finding from previous research (Kitayama and Markus, 2000; Kitayama et al., 2006) that disengaging emotions (e.g., anger) are more salient in Americans' emotional lives while engaging emotions (e.g., shame) prevail in Japanese emotional lives.

We have recently replicated these findings with samples of Japanese and Turkish students (Boiger et al., in preparation). Again, participants from both cultures rated, for most students they know, the frequency of anger and shame situations that had previously been sampled in Japan and Turkey. As before, we found that anger-eliciting situations were perceived to occur rarely in Japan, while shame-eliciting situations were perceived to occur frequently; this is in line with the Japanese goals of harmony maintenance and self-improvement. In Turkey, both anger and shame situations were perceived to occur frequently. This concurrent “up-regulation” of anger and shame situations may be typical for an honor-based interdependent cultural context, such as Turkey. In honor cultures, “honor must be claimed, and honor must be paid by others. A person who claims honor but is not paid honor does not in fact have honor” (Leung and Cohen, 2011, p. 509). The need to take a stand and uphold a reputation of toughness, while at the same time having to rely upon others to confirm one's reputation may explain the concurrent promotion of anger (as an emotion that helps in claiming honor) and shame (as an emotion that helps in preventing the withdrawal of honor through others) in Turkey. In comparison, in face-cultures, such as Japan, face cannot be claimed but is obtained by social conferral only; this explains why shame-promoting, but not anger-promoting situations were perceived as frequent in Japan.

### Appraisal

Mary may take John's rude behavior as a sign of his stress instead of blaming him for being offensive. This would be an example of emotion regulation through appraisal—in this case, down-regulation of anger. We review evidence that there are cultural differences in the prevalent types of appraisal that can be understood from the culturally valued relationships. Thus, when the Utku Inuit have a low tendency to blame, and this fact can be understood from their concern for avoiding confrontations, we assume that some kind of regulation is at play. At the level of the individual, it is often hard to know whether this regulation happens immediately (as when the Utku Inuit recognize less entitlement; Solomon, 1978), or whether it is a correction of an initially different response (as when they consider the mitigating circumstances). At the level of relationships, regulation more often takes the shape of re-appraisal, in particular when parents provide children with a different perspective on the emotional situation. Finally, structural conditions of everyday life may afford certain appraisals over others.

#### Individual tendencies

People's beliefs about the world will guide their appraisals. For example, whether the world is felt to be a predictable and controllable place might lead to different evaluations of events than when it is felt to be rather unpredictable and uncontrollable. Moreover, the appraisal dimension of controllability tends to be central in the appraisal patterns of anger and frustration (e.g., Frijda, 1986; Frijda et al., 1989; Stein et al., 1993; Kuppens et al., 2003): Experiencing anger implies that something has happened that is inconsistent with your goals, and that the situation is fixable and controllable. Therefore, one might expect cultural differences in the frequency and intensity of anger and frustration depending on the cultural schema of the world as controllable or uncontrollable. This expectation was confirmed by two studies in which European Americans' emotional responses were compared to those of Indians (Roseman et al., 1995) and Tahitians (Levy, 1978). Whereas the European American cultural ideals tend to emphasize control and predictability and, as such, promote a view of the world as malleable (Weisz et al., 1984; Mesquita and Ellsworth, 2001; Morling et al., 2002), Indian cultural ideals don't show this tendency (Miller et al., 1990; Savani et al., 2011). Consistently, Roseman and colleagues found that Indian college students rated self-reported emotional events to be less “incongruent with their motives” and reported lower overall intensities of anger than did their European American counterparts. Moreover, anger intensity was fully mediated by a person's perception of the event as discrepant with his or her goals. Similarly, the anthropologist Robert Levy pointed to the Tahitians' “common sense that individuals have very limited control over nature and over the behavior of others” (Levy, 1978, p. 226), and related this fact to the observation of a near absence of anger among the Tahitians. His explanation for this phenomenon was that a universe that is defined as unpredictable and uncontrollable might be “*cognitively* less frustrating than […] [a universe] in which almost anything is possible to individuals” (p. 226).

Cultural contexts also differ substantially with regard to the attribution of success or failure. European Americans have a pervasive tendency to attribute success to themselves, and failure to others or the situation; the opposite is true for East Asians (e.g., Heine et al., 1999). A recent study tested the idea that cultural differences in the appraisal of causal agency are associated with different emotional experiences (Imada and Ellsworth, 2011). Japanese and European American college students were asked to remember success and failure situations, to indicate if these situations had been caused by themselves, others, or circumstances, and to rate the intensity of their feelings. As expected, European Americans took more personal credit for success than the Japanese; Japanese credited circumstances for success. In contrast, the Japanese took more blame for failure than the European Americans; European Americans blamed others. These different appraisals were reflected in the emotions that the participants experienced: European Americans reported to feel pride when they succeeded, and anger or bad luck when they failed; Japanese reported to feel lucky after when they succeeded and shame when they failed. This pattern of success and failure attributions is consistent with the observed self-enhancing tendency that is characteristic of European American contexts and the tendency to focus on self-improvement characteristic of interdependent Japanese contexts. Moreover, the combined findings support the idea that people's habitual appraisals differ across cultures in ways that make culturally valued emotional experiences more likely.

There are also cultural differences in the perspective taken on situations: European Americans tend to take a first person perspective, but East Asians more readily emphasize the meaning of emotional situations for third others. These differences in perspective are likely to produce differences in emotional experience. For example, a first-person perspective on a situation may highlight how an event is inconsistent with one's goals, how others are responsible, and how others should accommodate to one's own wishes–all appraisals that render the experience of anger and frustration more likely (Frijda, 1986). In a comparison of European American and Japanese respondents, (Mesquita et al., unpublished) found that Japanese respondents reported indeed more appraisals that reflected an awareness of the meaning of the situation for other people. This study consisted of standardized interviews in which participants reported on their emotional experiences during a number of situations, e.g., situations of offense. Respondents reported a situation from their past, and their emotion narratives were recorded and later coded. The narratives suggested that, in the negative situations in particular, Japanese considered the meaning of the events for *other* people. This outside-in perspective on situations may be understood from the need to be socially attuned. For example, more than 40% of the Japanese, versus none of the European Americans, explained an offense situation from the perspective of a third person or a generalized other. In addition, in the offense situation, 56% of the Japanese compared to only 5% of the European Americans tried to understand or sympathize with the offender. Similarly, when Japanese adolescents were victim of another person's harmful behavior, they tended to make positive attributions of the other person's intentions or to engage in self-criticism (“She did not want to hurt me”; “Her behavior was accidental”; “I was wrong to give her the impression of my provocation”). Kornadt (2011), as reported in Trommsdorff (2012).

That cultural difference in perspective or appraisal lead to different emotional experiences is also suggested by a study in which people were asked to think about a past emotional event (Grossmann and Kross, 2010, Study 2). In this study, European American students reported more emotional distress and blame, which might give rise to more anger, than their Russian counterparts. These associations were partially mediated by cultural differences in the students' self-reflexive strategies about the event. European Americans recounted the emotionally arousing details of the past experience, thus immersing themselves in a first person perspective. In contrast, the more interdependently oriented Russians adopted a self-distancing perspective, thus imagining what the event could have meant to other people.

Whether people appraise situations to be about self-focused concerns or about their relationship with others, has implications for the types of emotions that they are likely to experience. In two studies with Belgian college students, participants described a recently experienced emotional situation and rated the intensity of a wide range of emotions during this situation (De Leersnyder and Mesquita, in preparation). They also indicated *if* and *to what extent* the situation had been either consistent or inconsistent with a number of different concerns that were based on the Schwartz value questionnaire (Schwartz, 1992). Some of these concerns were *other-focused* (e.g., Benevolence, Universalism and Conformity-Tradition), others were *self-focused* (Self-direction and Achievement). Across both studies, the concerns or values that were appraised as relevant to the situation predicted the type of emotions experienced. In situations that were relevant to other-focused values, the odds of experiencing socially engaging emotions were much higher than the odds of experiencing socially disengaging emotions. The opposite pattern of associations held for situations that were relevant to self-focused values. Moreover, the frequency with which these values were perceived as relevant to students' emotional situations exactly mirrored young Belgians' value hierarchy (i.e., most important values as “guiding principles in people's life”), as obtained from a national representative sample by the European Social Survey (ESS round 5; Norwegian social Science Data Services, 2012). This finding suggests that emotional experiences are culturally regulated to be *about* the most important cultural values: (1) culturally salient values are more readily available as standards of evaluation for emotional situations, and (2) the different types of values—self-focused vs. other-focused—translate into different patterns of emotional experience (more disengaging vs. engaging, respectively).

#### Relational co-regulation

Other people's appraisals are often referenced when people have to assess the meaning of situations (Parkinson and Simons, 2009). “Social referencing” is particularly evident in children, who often look at their caregivers' facial expressions when trying to appraise a situation as, for example, dangerous or safe (e.g., Campos and Stenberg, 1981); they can thus be said to construct the emotional meaning of the situation in conjunction with their caregivers (see Boiger and Mesquita, 2012). There is some evidence for cultural differences in the ways caregivers help their children (re-)appraise situations.

Different strategies for dealing with angry or frustrated children have been observed for European (American) and Japanese parents. One finding that stands out is that Japanese caregivers reason with their angry children, emphasizing how *others* feel when they hurt them (e.g., Conroy et al., 1980). Japanese parents thus helped the children adopt the outside-in perspective that is also common for Japanese adults. Re-appraising angering situations in this way may explain the lower levels of anger in Japan (Zahn-Waxler et al., 1996). Japanese parents rarely express direct disagreement with their non-compliant children; instead, they go through cycles of mutual perspective taking (Trommsdorff and Kornadt, 2003) or express negativity indirectly, e.g., by “suffering” (DeVos, 1985) or through silence (Johnson, 1995). By not providing direct verbal cues, parents give their children reason to consider circumstantial features of the event and to adjust their emotional response accordingly. In general, many of those parental regulatory strategies may increase empathy and heighten self-conscious emotions such as shame or guilt (Zahn-Waxler et al., 1979). European American parents, on the other hand, expect their children to self-assert and to stand up for themselves (Hess et al., 1980). When dealing with a non-compliant child, they tend to use more coercion (Conroy et al., 1980; Hess et al., 1980), e.g., removing the child from the situation. Similarly, in an (independent) German context, parents' behaviors encouraged appraisals of frustration in the child, leading to high levels of anger, and possibly to an escalation of the parent-child conflict (Trommsdorff and Kornadt, 2003). In independent contexts, parents tended to emphasize a first-person perspective on situations that may intensify the child's felt emotions (Cohen et al., 2007); a first person perspective also foregrounds socially disengaging emotions, such as anger (see also Harwood et al., 2002).

Co-regulation of appraisal also happens when parents pay attention to their children's emotions, and thus validate the appraisal of the situation, or to the contrary, ignore the child's emotions and fail to endorse the child's interpretation of the event. For example, German mothers who witnessed their children's mishaps focused on the children's distress, thereby confirming that the children had a good reason for their negative emotions. By contrast, Japanese and Indian mothers ignored their child's negative emotions, thus challenging their interpretation of the situation as one of distress (Trommsdorff and Friedlmeier, 1993, 2010; Trommsdorff, 2006).

Similarly, Cole and colleagues investigated how parents respond to their children's emotions in a series of studies with children from two Nepali ethnic groups–the Tamang and Brahman (Cole and Tamang, 1998; Cole et al., 2006). Although these two ethnic Nepali groups share core cultural values of interdependence, they emphasize different relational engagements. The Tamang—Tibetan Buddhists—emphasize egalitarianism, self-effacement and social harmony. The Tamang understand anger as a forceful emotion that interferes with the social goals of sharing and compassion, while shame is seen as a valuable emotion that implies the awareness of one's actions through the eyes of others. The Brahmans, on the other hand, are members of a high-status Hindu caste which is associated with ethnic pride, social dominance, and a high level of self-control. In Brahman eyes, anger constitutes a justifiable experience of a proud high-caste member that, nevertheless, needs to be regulated. Shame, on the other hand, is seen as a sign of personal weakness. Caregivers' responses to anger and shame episodes of 3- and 5-year old children differed accordingly between the groups. While Tamang caregivers reacted to expressions of anger by distraction and reasoning, Brahman caregivers paid more positive attention to anger episodes, supporting their children's appraisal that anger is justified. During shame episodes, Tamang caregivers responded with reasoning and nurturing, while Brahman caregivers largely ignored signs of shame, thus conveying that experiencing and displaying shame is not desirable. In these studies, caregivers appeared to co-regulate their children's emotion by helping them (re)-appraise the situation in ways that reinforce those emotions that are desirable according to their prevalent cultural ideals.

To our knowledge, there is no systematic empirical evidence that adults help each other in re-appraising situations in ways that are consistent with their cultural values. However, there is some anecdotal evidence for these co-regulatory processes beyond childhood. For instance, Kitayama and Masuda (1995) describe how US friends help each other when one is feeling shameful and down: “good friends are supposed to […] encourage the person by reorienting the person's attention away from his own shortcomings to external objects or events the person can reasonably blame for the impeding problem” (p. 220). These co-regulatory efforts may explain why shame is frequently transformed into anger in the American cultural context (Tangney et al., 1992, as cited in Kitayama and Masuda, 1995). By re-appraising the shameful event as caused by others rather than by oneself, the focus shifts from one's own painful shortcomings to the more empowering experience of self-integrity, and others' blameworthiness. Maintaining high self-esteem and avoiding self-critical information constitute central goals for the American independent self anger can thus be seen as a more desirable end-point of emotion regulation than shame.

#### Structural conditions

Finally, it is possible that an individual's environment is structured in ways that emphasize certain meanings or appraisals over others; a person's appraisal of the situation would thus depend on features of the situation that exert their influence independent of (or in interaction with) individual tendencies and relational co-regulation. Again, we would expect that these structural conditions emphasize appraisals that contribute to emotional experiences in line with the culturally defined end-points of emotion regulation.

In an impressive demonstration of the effect, Savani and colleagues (2011) have shown that participants apply another culture's interpretational schemes after having been exposed to a large number of situations from that culture. In this experiment, Savani and colleagues (2011, Study 5) exposed Indian and European American students to interpersonal situations that were sampled from both India and the US. As expected, Indian situations afforded more adjustment, whereas American situations afforded more influence. While the Indian participants reported initially more adjustment, and the US participants reported initially more influence, this pattern changed after the participants had been exposed to a large number of situations from both cultures; after 100 trials, the degree of adjustment reported by European American and Indian participants converged. Thus, both situational affordances (i.e., Indian situations call for accommodation) and individual psychological tendencies (i.e., Indians are by default more likely to adjust) contributed to cultural differences in how people reacted. While this study did not speak to the emotions that people experience, it does make a strong case for the idea that structural conditions afford certain appraisals, which in turn should be associated with different emotional experiences.

A direct investigation of how structural conditions across cultures afford certain appraisals over others, and thus regulate emotional experiences, does not exist to our knowledge; however a few first promising results from a monocultural study point in this direction. Kuppens et al. (2008) showed that people's appraisal of angering situations depends to a large extent on the antecedent situations themselves. In two studies, situational differences were a predictor (above and beyond individual differences) of the types of appraisals used, accounting for about 20% of the variance in individual responses; in the words of the authors, “different circumstances can pull for different characteristic appraisals” (p. 10). Although their data were collected among Belgian (Dutch-speaking) participants only, these results clearly speak to the importance of situational characteristics for individual emotional experience.

## Conclusion

Emotional experience tends to be aligned with the culturally valued ways of relating. This alignment can be attributed to emotion regulation—i.e., all processes that help to attain the culturally appropriate emotional experiences. In this article we have reviewed the evidence for antecedent-focused emotion regulation; that is regulation at the time of emotion elicitation. We focused on two types of emotion regulation that fall under this category: situation selection and appraisal. We discussed research showing cultural differences in situation selection and appraisal at the level of the individual, the relationship, and the structure of everyday life. The combined evidence suggests that much of the cultural regulation of emotions takes place at the start of the emotion process, before there even is an emotional experience. Response-focused emotion regulation, in the form of suppression of emotional experience or expression, may be only one of the many types of cultural regulation of emotions (e.g., Matsumoto et al., 2008). In fact, we submit that cultural regulation is *most likely* to target the elicitation of emotions itself, since suppression of already activated resources is much more effortful. Response-focused regulation may be “culture's last resort” for shaping emotions in a culturally normative fashion–only to be used when all other ways failed.

Our cultural perspective on emotion regulation highlights that emotion regulation is not merely an *intrapersonal* process. Rather, emotions are also regulated by others in our environment, and by the ways in which our social worlds are structured in terms of both and furthermore by adopting a cultural perspective we highlighted differences in the “endpoints” of emotion regulation, even if emotion regulation universally aims to improve the individual's social adjustment. Finally, a cultural perspective underlines that much of emotion regulation often happens outside the awareness of the individual—through the situations that are culturally promoted and the appraisals that are condoned and activated. This means that most, if not all emotional experiences are (culturally) regulated to some extent, even the ones that appear “natural” to us.

### Conflict of interest statement

The authors declare that the research was conducted in the absence of any commercial or financial relationships that could be construed as a potential conflict of interest.

## Footnotes

^1^The idea that emotion regulation is functional, does not imply that regulation outcomes are adaptive in every situation. Rather, regulation shapes emotions in ways that are usually more effective within a given socio-cultural context than they would have been without regulation.
